# Supplementary material for: Comparison of Nucleosome Landscapes Between Porcine Embryonic Fibroblasts and GV Oocytes
Source: Animals (Basel). 2024 Nov 25;14(23):3392. doi: 10.3390/ani14233392 (PMC11840278; doi:10.3390/ani14233392)
Supplement: Supplementary file 1 [file animals-14-03392-s001.zip › animals-3205113-supplementary.pdf]

Table S1: Basic source information of the MNase-seq and RNA-seq data

Accession	Type	Samples	Platform	Type	Raw Reads (Mil.)	Tissue or cell (conditions)	reference
PRJNA347494	MNase-seq	3	HiSeq 2000	Pair (120 bp×2)	92.86	GV oocyte (pig)	8
SRX7575382	RNA-seq	3	HiSeq 2500	Pair (100 bp×2)	19.84	GV oocyte (pig)	-
SRP090055	MNase-seq	3	HiSeq 2000	Pair (120 bp×2)	209.19	porcine embryonic fibroblasts	2
SRX027095	RNA-seq	3	HiSeq 2000	Pair (100 bp×2)	53.06	porcine embryonic fibroblasts	-

Table S2: Basic quality and analysis information of the data

Sample	Oocyte		PEF	
(Average Value)	MNase-seq	RNA-seq	MNase-seq	RNA-seq
Raw reads (Mil.)	92.86	19.84	209.19	53.06
Clean reads (Mil.)	70.57	17.66	139.64	46.98
Error rate (%)	0.17_0.44	0.18_0.33	0.19_0.65	0.25_0.66
GC (%)	46.75	47.21	45.55	44.82
Q20 (%)	98.03	98.12	96.94	97.46
Q30 (%)	95.13	97.11	92.23	95.45
Uniquely mapped (Mil.)	49.62	14.67	114.00	37.68
Percentage of uniquely mapped (%)	70.32	83.06	81.64	80.28
cover rate of genome (%)	72.67	—	76.09	—

Table S3: Nucleosome ratios of oocyte and PEF in different functional elements. Related to Fig.1A and Fig. 1B.

Genomic Items	Genome_Ratio (%)	Nucleosoem_Ratio of GV oocyte average (BF, %)	Nucleosoem_Ratio of PEF average (PEF, %)
All	100	100	100
Promoter	1.07	4.85	3.3
5’ UTR	0.1	0.83	0.57
3’ UTR	0.49	2.69	1.89
First Exon	1.94	0.9	0.62
Other Exons		5.05	3.16
Introns	22.95	47.08	45.87
Intergenic Region	73.44	38.6	44.58

Table S4: The only expressed genes identified in BF and PEF by RNA-seq.

PEF		BF	
Gene ID	Gene Name	Gene ID	Gene Name
ENSSSCG00000004789	THBS1	ENSSSCG00000007691	ZP3
ENSSSCG00000010447	ACTA2	ENSSSCG00000010141	ZP4
ENSSSCG00000017082	SPARC	ENSSSCG00000004285	OOEP
ENSSSCG00000016174	FN1	ENSSSCG00000021959	SYCN
ENSSSCG00000036135	COL1A1	ENSSSCG00000004283	DPPA5
ENSSSCG00000016034	COL3A1	ENSSSCG00000007851	ZP2
ENSSSCG00000017723	CCL2	ENSSSCG00000009610	NPM2
ENSSSCG00000016035	COL5A2	ENSSSCG00000040478	RF02271
ENSSSCG00000004509	LIPG	ENSSSCG00000003307	NLRP9
ENSSSCG00000018303	RF00494	ENSSSCG00000015020	BTG4
ENSSSCG00000006472	CRABP2	ENSSSCG00000003484	PADI6
ENSSSCG00000020439	RF00009	ENSSSCG00000039560	OOSP2
ENSSSCG00000011033	VIM	ENSSSCG00000015101	FOXR1
ENSSSCG00000004241	GJA1	ENSSSCG00000016489	WEE2
ENSSSCG00000011973	COL8A1	ENSSSCG00000008824	ZAR1
ENSSSCG00000002795	CDH11	ENSSSCG00000024966	TRIM77
ENSSSCG00000036396	RF01956	ENSSSCG00000008321	FIGLA
ENSSSCG00000016233	SERPINE2	ENSSSCG00000007702	FKBP6
ENSSSCG00000033344	RF01973	ENSSSCG00000017883	GGT6
ENSSSCG00000014232	LOX	ENSSSCG00000014222	ARL14EPL
ENSSSCG00000009216	SPP1	ENSSSCG00000012198	MAGEB3
ENSSSCG00000004192	CCN2	ENSSSCG00000013363	LDHC
ENSSSCG00000005751	COL5A1	ENSSSCG00000015879	DAPL1
ENSSSCG00000036049	PEG10	ENSSSCG00000001535	TCP11
ENSSSCG00000025698	SERPINE1	ENSSSCG00000014222	ARL14EPL
ENSSSCG00000040642	LASP1	ENSSSCG00000031220	TEX35
ENSSSCG00000021084	S100A6	ENSSSCG00000015544	ZNF648
ENSSSCG00000005380	COL15A1	ENSSSCG00000035667	KBTBD13
ENSSSCG00000037614	RF01957	ENSSSCG00000013363	LDHC
ENSSSCG00000005316	TPM2	ENSSSCG00000006316	MAEL
ENSSSCG00000038447	RF01295	ENSSSCG00000007703	TRIM50
ENSSSCG00000016634	CAV1	ENSSSCG00000039218	SLC38A8
ENSSSCG00000005267	ANXA1	ENSSSCG00000040408	SAXO1
ENSSSCG00000005287	PSAT1	ENSSSCG00000013503	SHD
ENSSSCG00000008294	ACTG2	ENSSSCG00000012133	ASB11
ENSSSCG00000013614	CNN1	ENSSSCG00000014271	MEIKIN
ENSSSCG00000020976	CSRP1	ENSSSCG00000012198	MAGEB3
ENSSSCG00000033608	LOXL2	ENSSSCG00000007382	PABPC1L
ENSSSCG00000009668	CLU	ENSSSCG00000036367	PABPN1L
ENSSSCG00000035392	IGFBP2	ENSSSCG00000003314	NLRP11
ENSSSCG00000031503	PRRX1	ENSSSCG00000001847	MESP1
ENSSSCG00000010517	PGAM1	ENSSSCG00000010656	CCDC172
ENSSSCG00000025924	IGFBP5	ENSSSCG00000016478	PRSS2
ENSSSCG00000001565	CDKN1A	ENSSSCG00000023057	LMO1
ENSSSCG00000004484	COL12A1	ENSSSCG00000040814	IL28B
ENSSSCG00000036049	PEG10	ENSSSCG00000017556	ANKRD40CL
ENSSSCG00000025698	SERPINE1	ENSSSCG00000031324	ZYG11A
ENSSSCG00000040642	LASP1	ENSSSCG00000012310	BMP15
ENSSSCG00000021084	S100A6	ENSSSCG00000008394	FANCL

ENSSSCG00000005380	COL15A1	ENSSSCG00000004873	FBXO15
ENSSSCG00000037614	RF01957	ENSSSCG00000009089	ADAD1
ENSSSCG00000005316	TPM2		
ENSSSCG00000038447	RF01295		
ENSSSCG00000016634	CAV1		
ENSSSCG00000005267	ANXA1		
ENSSSCG00000005287	PSAT1		
ENSSSCG00000008294	ACTG2		
ENSSSCG00000013614	CNN1		
ENSSSCG00000020976	CSRP1		
ENSSSCG00000033608	LOXL2		
ENSSSCG00000009668	CLU		
ENSSSCG00000035392	IGFBP2		
ENSSSCG00000031503	PRRX1		
ENSSSCG00000010517	PGAM1		
ENSSSCG00000025924	IGFBP5		
ENSSSCG00000001565	CDKN1A		
ENSSSCG00000004484	COL12A1		
ENSSSCG00000036723	EMP1		
ENSSSCG00000023048	RF00017		
ENSSSCG00000033299	FLNA		
ENSSSCG00000005494	TNC		
ENSSSCG00000032330	THY1		
ENSSSCG00000011046	ITGA8		
ENSSSCG00000010461	ANKRD1		
ENSSSCG00000035077	INHBA		
ENSSSCG00000021978	-		
ENSSSCG00000015270	FMOD		
ENSSSCG00000040162	NUPR1		
ENSSSCG00000017089	ANXA6		
ENSSSCG00000017707	TAF15		
ENSSSCG00000006940	CCN1		
